# Supplementary material for: Switch to second-line versus continued first-line antiretroviral therapy for patients with low-level HIV-1 viremia: An open-label randomized controlled trial in Lesotho
Source: PLoS Med. 2020 Sep 16;17(9):e1003325. doi: 10.1371/journal.pmed.1003325 (PMC7494118; doi:10.1371/journal.pmed.1003325)
Supplement: S1 Statistical Analysis Plan — (PDF) [file pmed.1003325.s002.pdf]

DocuSign Envelope ID: C2EAC1E7-7B2D-4962-BED6-57122FAF0196

SESOTHO: Switch to second-line versus WHO-guided standard of care for unsuppressed patients on first-line ART with viremia below 1000 copies/mL – a multicenter, parallel-group, open-label, randomized clinical study in rural Lesotho

STATISTICAL ANALYSIS PLAN

Version 1.1, 21 April 2020

Trial registration: ClinicalTrials.gov, NCT03088241, 5 May 2017

Current protocol version: 15, dated 2 Nov 2018

(Statistical analysis plan contents following Gamble et al (1))

DocuSign Envelope ID: C2EAC1E7-7B2D-4962-BED6-57122FAF0196

1. Administrative information

Revision history

Version	Date	Changes and reasons
0.1	-	Summary in the protocol
0.2	13 February 2020	First draft
0.3	12 March 2020	Revised throughout following feedback from all persons listed below
0.4	19 March 2020	Revised throughout following further feedback from Alain Amstutz and Niklaus Labhardt
1.0	2 April 2020	Revised following final feedback from Alain Amstutz; final version for signature
1.1	21 April 2020	Correction to analysis visit windows (Table 2) further to feedback from sites

Roles and responsibilities

Name	Affiliation	Role
Niklaus Labhardt	Swiss TPH	Principal investigator
Josephine Muhairwe	SolidarMed Lesotho	Local principal investigator
Bienvenu Nsakala	SolidarMed Lesotho	Study physician
Alain Amstutz	Swiss TPH	Co-investigator
Fiona Vanobberghen	Swiss TPH	Trial statistician
Tracy Glass	Swiss TPH	Supervising statistician

Signatures

Name	Signature	Date
Fiona Vanobberghen	DocuSigned by: Fiona Vanobberghen 99EE806461C740F...	4/21/2020
Niklaus Labhardt	DocuSigned by: Niklaus Labhardt 43A25571C7C149F...	4/21/2020

DocuSign Envelope ID: C2EAC1E7-7B2D-4962-BED6-57122FAF0196

Abbreviations

AE	Adverse event
ALT	Alanine transaminase
ART	Antiretroviral therapy
AST	Aspartate transaminase
BMI	Body mass index
CI	Confidence interval
CRF	Case report form
CTCAE	Common terminology criteria for adverse events
INSTI	Integrase strand transfer inhibitor
IQR	Interquartile range
LLN	Lower limit of normal
LTFU	Lost to follow-up
NNRTI	Non-nucleotide reverse transcriptase inhibitor
SAE	Serious adverse event
SE	Standard error
ULN	Upper limit of normal
VL	Viral load

Data management and sharing

Refer to the document “SESOTHO codebook” which describes the data capture, entry, and query resolution process.

Key trial data will be made available before publication of the main manuscript through an appropriate data repository such as Zenodo, and will be referenced accordingly in the main manuscript.

DocuSign Envelope ID: C2EAC1E7-7B2D-4962-BED6-57122FAF0196

Contents

1. Administrative information 2
Revision history 2
Roles and responsibilities 2
Signatures 2
Abbreviations 3
Data management and sharing 3
2. Introduction (from protocol synopsis) 6
2.1 Background and rationale 6
2.2 Objectives 6
3. Study methods 7
3.1 Trial design 7
3.2 Randomisation 8
Table 1. Sites 8
3.3 Sample size 9
3.4 Framework 9
3.5 Statistical interim analyses and stopping guidance 9
3.6 Timing of final analysis 9
3.7 Timing of outcome assessments 9
Table 2. Nominal visits and permitted windows. 9
4. Statistical principles 10
4.1 Confidence intervals and p-values 10
4.2 Adherence and protocol deviations 10
4.3 Analysis populations 10
5. Trial population 10
5.1 Screening data 10
5.2 Eligibility 10
5.3 Recruitment 10
5.4 Withdrawal/follow-up 11
5.5 Baseline patient characteristics 11
6. Analysis 11
6.1 Outcome definitions 11
Table 3. Laboratory parameter categorisations as per CTCAE. 12
6.2 Analysis methods 13
6.3 Missing data 15

DocuSign Envelope ID: C2EAC1E7-7B2D-4962-BED6-57122FAF0196

6.4 Additional analyses ... 15
6.5 Harms ... 15
6.6 Statistical software ... 16
7. References ... 16
8. Shell tables ... 17
Table 8.1. Baseline characteristics by group: demographics. ... 17
Table 8.2. Baseline characteristics by group: clinical. ... 19
Table 8.3. Baseline characteristics by group: laboratory. ... 20

DocuSign Envelope ID: C2EAC1E7-7B2D-4962-BED6-57122FAF0196

2. Introduction (from protocol synopsis)

2.1 Background and rationale

The Joint United Nations Programme on HIV/AIDS (UNAIDS) launched the 90-90-90 targets for 2020 based on the result of newly-acquired scientific evidence that – irrespective of CD4 count – early antiretroviral treatment (ART) for HIV-positive individuals is beneficial to them and prevents HIV transmission. UNAIDS expects that the 90-90-90 targets will lead to a reduction in the yearly global HIV incidence from 2 million currently to 500,000 by 2020.

A crucial step to achieve the third pillar of the UNAIDS 90-90-90 targets – 90% viral suppression among HIV-positive individuals on treatment – and thus ensure a successful treatment outcome is the monitoring and management of first-line ART failure.

Since 2013, the WHO recommends routine viral load (rVL) measurement as the preferred monitoring strategy in resource-limited settings and defines virological failure as confirmed VL ≥ 1000 copies/mL despite good adherence. Specifically, the guidelines recommend that in case of a VL ≥ 1000 copies/mL the patient should undergo enhanced adherence support and a second VL test 3 months later. A second VL ≥ 1000 copies/mL with confirmed good adherence would trigger the switch to a second-line regimen, whereas if the VL is < 1000 copies/mL the patient should continue unaltered first-line ART. However, the optimal threshold for defining virological failure and the need for switching ART regimen has not yet been determined. In fact, people with VL levels of less than 1000 copies/mL, but not fully suppressed (usually defined as 50-100 copies/mL), are at a increased risk for drug resistance mutations (DRM) and subsequent virological failure. A recently published study from our research consortium in Lesotho indicates similar findings, demonstrating a significant accumulation of drug resistance mutations in patients with VL levels of less than 1000 copies/mL.

The VL threshold of 1000 copies/mL recommended by the WHO and the Lesotho national guidelines for the switch to second-line ART is likely to miss a substantial number of patients on first-line ART with persisting virus replication below 1000 copies/mL with DRM. In resource-limited settings where VL monitoring is not as frequent as in high-income countries, this could have serious implications: after a VL below 1000 copies/mL the patient may not receive a follow-up VL for up to a year, and thus may continue on a failing regimen for a long period of time. In conclusion, such patients are at increased risk for DRM, accumulation of further resistance mutations, drug-resistant virus transmission, and subsequent virological failure.

We hypothesize that in patients on first-line ART with two consecutive unsuppressed VL measurements ≥ 100 copies/mL, where the second VL is between 100 and 999 copies/mL, switch to second-line ART (intervention group) will lead to a higher rate of viral resuppression (VL < 50 copies/mL) and is therefore superior compared to not switching to second-line ART according to WHO guidelines (control group, standard of care).

2.2 Objectives

The objective of the trial is:

- To challenge the WHO VL threshold for treatment failure (1000 copies/mL) for HIV positive individuals on first-line ART in a resource-limited setting.

DocuSign Envelope ID: C2EAC1E7-7B2D-4962-BED6-57122FAF0196

3. Study methods

3.1 Trial design

Full details of the trial are available in the published protocol (2).

This is a multi-centre, two-arm, parallel-group, open-label randomised controlled trial, conducted in health facilities in Lesotho. Eligible persons are HIV-positive individuals on non-nucleoside reverse transcriptase inhibitor (NNRTI) based first-line ART (standard first-line ART in Lesotho) for at least 6 months with reported good adherence, attending one of the study sites, and with two consecutive unsuppressed VL ≥100 copies/mL with the second VL between 100 and 999 copies/mL. Full inclusion/exclusion criteria are as follows:

Inclusion criteria:

- On first-line ART with two consecutive unsuppressed VL ≥ 100 copies/mL, with the second VL between 100 and 999 copies/mL
- Lives and/or works in one of the study districts and intends to seek follow-up at one of the study facilities
- Signed written informed consent. For children aged <16 years, a main caregiver, and for illiterate a literate witness, has to provide oral and written informed consent

Exclusion criteria:

- On ART less than 6 months
- On protease-inhibitor containing ART or any other second-line ART
- Poor adherence (self-reported at least 1 dose missing in the last 4 weeks, respectively 2 doses of a twice-daily-regimen)
- Clinical WHO stage 3 or 4 at enrolment

Randomisation is in a 1:1 ratio to intervention (switch to second-line ART) or control (no switch to second-line ART). Follow up is over 9 months (for primary endpoint and study completion), with extended follow up over 24 months.

The primary outcome of the trial is:

- Proportion of virologically suppressed (VL < 50 copies/mL) participants at 9 months

The secondary outcomes of the trial are:

- Proportion of participants with different VL thresholds (VL <100, <200, <400, <1000 copies/mL) at 9 months
- Proportion of participants with viral resuppression (<50 copies/mL) at 6 months
- Sustained virologic failure: Proportion of participants with unsuppressed VL >50 copies/mL at 6 and 9 months
- Adherence at 3, 6, 9 months, assessed by self-reported missed doses
- Clinical outcomes at 9 months: change from baseline in body weight, haemoglobin, CD4 count, lipids (total cholesterol, HDL cholesterol, LDL cholesterol, triglycerides); proportion

DocuSign Envelope ID: C2EAC1E7-7B2D-4962-BED6-57122FAF0196

of participants with new clinical WHO 3 or 4 events; proportion of participants died (all-causes)
- Proportion of patients with any adverse events (AEs) or serious adverse events (SAEs) at 9 months
- Long-term follow-up endpoint: Proportion of patients that are alive, retained in care and virologically suppressed (VL < 50 copies/mL) at 24 months

Exploratory outcomes are:

- Cost-effectiveness
- Prevalence of major drug resistance mutations a) on all baseline VLs and b) on all VLs that remain unsuppressed (>50 copies/ml) at 9 months for all samples for which an RT-PCR amplification is successful

Effect modification of the primary outcome will be assessed by:

- demographic groups (children [defined as <16 years old at enrolment] vs pregnant women vs adults)
- VL group at enrolment (second VL 100-599 vs 600-999 copies/mL copies/mL)

A subgroup analysis for the primary outcome will be done among:

- Individuals showing a >0.5 drop in log10 VL from the first to the second VL measurements pre-randomization.

3.2 Randomisation

Randomisation was stratified by demographic groups (children versus pregnant women versus adults); VL groups at enrolment (second VL 100-599 versus 600-999 copies/mL); and site (see Table 1; health centres combined, and Butha-Buthe and Mokhotlong Hospitals combined for logistical reasons), using randomly-varying block sizes (details on block sizes held by trial statistician). The randomisation list was generated by computer by the trial statistician, who was not involved in the recruitment or follow-up of participants.

Table 1. Sites.

Sites	District	Type of facility
Butha-Buthe Government Hospital	Butha-Buthe	Hospital
Seboche Mission Hospital	Butha-Buthe	Hospital
St Paul Health Center	Butha-Buthe	Health centre
St Peters Health Center	Butha-Buthe	Health centre
Muela Health Center	Butha-Buthe	Health centre
Mokhotlong Hospital	Mokhotlong	Hospital
Senkatana Hospital	Maseru	Hospital
Motebang Hospital	Leribe	Hospital

Randomisation was performed using sealed, opaque and sequentially-numbered envelopes, which were prepared by person(s) independent to the trial.

DocuSign Envelope ID: C2EAC1E7-7B2D-4962-BED6-57122FAF0196

The first five randomisations in each stratum were checked by the statistician as soon as possible after randomisation to ensure that the procedures were working correctly; no errors were detected.

3.3 Sample size

Based on data from previous research which we conducted in Lesotho, we expect 9-month viral resuppression rates of 25% in the control arm and 60% in the intervention arm, assuming a LTFU-rate of 10% in each arm and assuming such participants as failure (=virologically not suppressed). Assuming a two-sided type 1 error of 5% and a power of 90%, 80 individuals (40 per group) are needed to detect a 35% difference. With this sample size we will still yield 78% power to detect a 30% difference. With an estimated 10% trial participation refusal rate, we need to screen approximately 90 participants.

3.4 Framework

This is a superiority trial.

3.5 Statistical interim analyses and stopping guidance

No interim analyses are planned.

3.6 Timing of final analysis

Outcomes will be analysed after the last participant has completed their primary outcome visit at 9 months. Long term follow-up will continue until the last patient has completed their 24 month follow-up, after which time the long term follow-up analyses will be performed.

3.7 Timing of outcome assessments

Table 2 shows the nominal visit months, the permitted ranges according to the protocol, and the ranges that will be used for analysis. One month is defined as 28 days (i.e., 4 weeks), therefore the analysis windows are defined in terms of weeks. The lower limits of the analysis windows correspond to the lower protocol-defined limit (using 28 days per month and converted to weeks). Some sites used calendar months to calculate follow-up visit dates, therefore the upper windows for analysis allow for this (plus a leeway of approximately two weeks, and ensuring that the analysis windows “touch” where possible so that all visits are assigned to a nominal visit month).

Table 2. Nominal visits and permitted windows.

Months		Conversion to weeks, with 28 days per month		Analysis window, weeks
Nominal visit month	Range according to the protocol, months	Nominal visit week	Range according to the protocol, weeks	
3	2-4	12	8-16	>=8 and <20
6	5-7	24	20-28	>=20 and <32
9	8-10	36	32-40	>=32 and <52
24	22-26	96	92-100	>=88 and <130

DocuSign Envelope ID: C2EAC1E7-7B2D-4962-BED6-57122FAF0196

4. Statistical principles

4.1 Confidence intervals and p-values

Statistical tests and confidence intervals will be two-sided. All estimates will be presented with 95% confidence intervals. P-values will be presented where appropriate. No adjustments will be made for multiple testing; interpretations will be based on the strength of evidence of effect size and consistency of results for related outcomes.

4.2 Adherence and protocol deviations

Immediately following randomisation, participants randomised to the control group are expected to remain on first line ART, and participants randomised to the intervention group are expected to switch to second line ART. We will present the number of participants on second line ART after randomisation and at each scheduled follow-up visit, by randomised group (with the proportion of those who attended the given visit). We will further tabulate the ART regimens at each scheduled visit, by group.

Adherence to the ART prescribed is one of the secondary outcomes, and is described in section 6.

Any protocol deviations as reported by the trial team or detected at the time of data cleaning/analysis will be described by group.

4.3 Analysis populations

Analyses will be by intention to treat, that is including all participants as randomised.

In sensitivity analyses for the primary outcome, we will also consider a “per protocol set” which is further defined in section 6.2.

5. Trial population

5.1 Screening data

No specific screening data were captured, aside from those used to determine eligibility for the trial (see sections 3.1 and 5.2).

5.2 Eligibility

Screening/eligibility data will be summarised in a CONSORT flowchart, showing the total number of people screened and the reasons for screening failures as per the eligibility criteria in section 3.1.

5.3 Recruitment

The CONSORT flowchart will include the numbers of participants randomised by group. Enrolment will also be presented by the stratification factors. The two viral loads that are required for determining eligibility will be summarised. A pre-specified subgroup of interest is those participants

DocuSign Envelope ID: C2EAC1E7-7B2D-4962-BED6-57122FAF0196

who had >0.5 log drop between the two screening VLs, therefore enrolment of such participants will also be presented.

5.4 Withdrawal/follow-up

The CONSORT flowchart will summarise follow-up for each of the scheduled visits, by randomised group. Reasons will be given for participants who did not complete follow-up as expected. We will also present time from randomisation to last follow-up (categorised as <12, 12-<24, 24-<36, 26-<96, ≥96 weeks).

5.5 Baseline patient characteristics

Baseline characteristics will be summarised by randomised group, using medians and interquartile ranges for continuous variables and numbers and percentages for categorical variables. Shell tables showing the variables and categories are included in section 8. Assessment will be made for baseline imbalances between the randomisation groups by visual inspection only, by the trial team before looking at outcome data. In sensitivity analyses, we will further adjust the outcome analyses for any covariates so identified (erring on the side of inclusivity). There will be no formal testing of baseline characteristics across randomised groups (3,4).

6. Analysis

The screening visit and randomisation are expected to happen on the same date. Time will be measured from randomisation.

The “baseline” viral load result is defined as that closest to randomisation, with a window of -14 weeks to 0 days relative to the date of randomisation. Most participants are expected have a baseline VL result up to a maximum of 4-8 weeks before randomisation, but we allow up to 14 weeks for the few patients whose next regular visit was scheduled up to three months later, and it was not possible to get the patient to return sooner. This was anticipated and allowed for in the protocol. For all other laboratory results, the “baseline” result is defined as that closest to randomisation, with a window of -14 days to +14 days relative to the date of randomisation. We will present data summarising the timing of baseline viral load and other laboratory results relative to the date of randomisation. The same window of -14 to +14 days will be used for defining baseline clinical measurements such as weight and blood pressure.

There will be no independent programming of the primary outcome, rather we rely on the experience of the team for the accuracy of the data, analyses and interpretations; and the results will be assessed as a whole for consistency.

6.1 Outcome definitions

The primary and secondary outcomes based on VL categories are as defined in section 3.1. For participants who did not have VL measured within the required window (including due to LTFU or death, or had VL measured but had changed regimen line due to clinical failure [see below]), VL are considered as missing.

DocuSign Envelope ID: C2EAC1E7-7B2D-4962-BED6-57122FAF0196

Change in regimen line due to clinical failure is defined as a change to a new drug class, e.g. from NNRTI to protease inhibitor, due to clinical failure defined by a new WHO 3 or 4 event. Therefore substitutions for side-effects, e.g. changing from tenofovir to zidovudine because of renal impairment, are not considered change in regimen line. Further, changes to regimen line due to other reasons, for example due to stock outs, are not included in this definition. There are anticipated to be few participants changing regimen line due to clinical failure or stock outs.

“>0.5 log drop between screening VLs” is defined as those meeting the following criterion:
[Log10(second to last VL) – log10(last VL)] >0.5.

The secondary outcome of adherence will be assessed by self-reported doses missed. We define participants reporting having missed no doses in the last 4 weeks as having good adherence.

BMI will be calculated as weight in kg divided by height in m^2, and categorized according to WHO classifications of underweight (<18.5 kg/m^2), normal (18.5-<25 kg/m^2) and overweight/obese (≥25 kg/m^2) (5).

As per the protocol, AE and SAE are graded by the study staff according to CTCAE (version 4, published 28 May 2009) (6).

In addition to the AEs reported, we will assess the laboratory measurements of ALT, AST, haemoglobin and creatinine (after conversion to estimated glomerular filtration rate (eGFR)) according to CTCAE grades 3 and 4 (Table 2). eGFR will be calculated using the Cockcroft-Gault equation (7):

$$eGRF = \frac{(140 - age) \times (weight\ in\ kg) \times constant}{serum\ creatinine\ in\ micromol/L}$$

where constant = 1.23 for men and 1.04 for women, and assessed as in Table 2.

Table 3. Laboratory parameter categorisations as per CTCAE.

Laboratory measurement	Adverse event in CTCAE	Grade 3	Grade 4	ULN Mokhotlong, Paballong, Seboche, Motebang laboratories	ULN national reference laboratory (Senkatana)
ALT (IU/L)	“Alanine aminotransferase increased”	>5.0*ULN to 20.0*ULN	>20.0*ULN	40	31
AST (IU/L)	“Aspartate aminotransferase increased”	>5.0*ULN to 20.0*ULN	>20.0*ULN	37	31
Haemoglobin (g/dL)	“Anemia”	<8.0	-	-	
eGFR (ml/min/1.73m^2)	“Chronic kidney disease”	15-<30	<15	-	

ULN=upper limit of normal.

Major drug resistance mutations will be determined according to the Stanford University Resistance Database (8).

DocuSign Envelope ID: C2EAC1E7-7B2D-4962-BED6-57122FAF0196

6.2 Analysis methods

Continuous variables will be inspected using histograms: 1) to assess for outliers which may be queried for accuracy, and 2) to assess whether appropriate transformations are required for modelling.

Outcomes will be summarised using means and standard errors for continuous variables and numbers and percentages for categorical variables, by randomised group. Percentages will be reported to zero decimal places, unless <0.5% when they will be given to one decimal place.

We will present numbers and proportions of participants with VL below different thresholds (<50, <100, <200, <400, <1000 copies/m) at 9 months (and <50 copies/ml at 6 months), with those missing VL results within the required analysis windows as defined in section 3.7 (including due to LTFU, death or regimen line change due to clinical failure (defined in section 6.1)) counted as "failures". We will provide details about the reasons for missing VLs (i.e., LTFU for that visit, died, regimen line change due to clinical failure).

For the analysis of the primary outcome, we will use a logistic regression model adjusted for the randomisation stratification factors (demographic groups (children vs pregnant women vs non-pregnant adults), VL at enrolment (second VL 100-599 vs 600-999 copies/mL)), but not centres since we have few participants at a number of centres. LTFU, death and regimen line change due to clinical failure prior to determination of the primary endpoint will be considered as failures. Results will be reported as odds ratios with 95% confidence intervals. Further, we will estimate the risk difference, with 95% confidence intervals estimated using the delta method (9).

In a sensitivity analysis for the primary outcome, we will analyse the per protocol set, considering only participants that finished the 9 months according to the protocol (=alive, retained in care, no change in regimen line other than that indicated by the randomisation (regardless of the reason), VL measurement available at 9 months). We will summarise the data (numbers and proportions of participants included in this analysis, and meeting the primary outcome), and fit logistic regression models as for the main primary outcome analysis.

In a further sensitivity analysis for the primary outcome, we will repeat the analyses using only VLs measured within the protocol-defined windows (summarising the data, and fitting logistic regression models as above).

For assessment of effect modification of the primary outcome by demographic groups and VL at enrolment (see section 3.1), analyses will be performed as for the main primary outcome analysis, and including in the models an interaction between the potential effect modifiers and randomised group, with recognition that power may be low. If there are small numbers of participants within certain subgroups (for example, children and/or pregnant women), then such an approach may not be feasible and instead we would restrict the model to the subgroups of sufficient size.

For assessment of the subgroup analysis (those with >0.5 drop in log10 VL), analyses will be performed as for the main primary outcome analysis, with the dataset restricted to individuals showing a >0.5 drop in log10 VL from the first to the second VL measurements pre-randomization.

For the secondary outcomes defined by different VL thresholds or time points, we will follow the same approach as for the main primary outcome analysis.

DocuSign Envelope ID: C2EAC1E7-7B2D-4962-BED6-57122FAF0196

For the outcome of sustained virologic failure, we will also follow the same approach as for the main primary outcome analysis. We will summarise the number and proportion of participants with both 6 and 9 month VL results, and whether <50 copies/ml at one or both. For participants missing either the 6 or 9 month VL (but not both), if the non-missing VL is <50 copies/ml then the participant will be considered NOT to have sustained virologic failure. In line with the main primary analyses, participants missing both 6 and 9 month VL results will be considered as failures (i.e., has sustained virologic failure). Similarly, participants missing either the 6 or 9 month VL and with the non-missing VL ≥50 copies/ml, then the participant will be considered as a failure. As for the main primary outcome analyses, "missing VL" here means no VL measured within the required analysis window (including due to LTFU or death, or regimen line change due to clinical failure).

For the adherence outcome, we will report the number and proportion of participants with good adherence (see definition in section 6.1) at each of the scheduled visits, by randomised group. To compare between groups, we will use a logistic regression model for each of these timepoints separately, adjusted for the same variables as the primary outcome analysis. The analysis of this outcome will be restricted to participants who attended and answered the adherence question at the respective follow-up visits.

We will also assess overall adherence over the whole trial, reporting the number and proportion of participants with good adherence at every one of the 3, 6 and 9 month visits, versus poor adherence at ≥1 of these three visits. For participants without adherence data at all three visits, if the participant had poor adherence reported for at least one of the visits then he/she will be included as having overall poor adherence; otherwise he/she will be excluded from the analysis. To compare between groups, we will fit a logistic regression model, adjusted for the same variables as the primary outcome analysis.

We will also consider a repeated measures analysis, using a mixed effects logistic regression model with the outcome of good adherence and each participant contributing up to three measurements (one from each of the three visits at 3, 6 and 9 months). Visit will be included in the model as a fixed effect, along with the same variables as the primary outcome analysis. Participant will be included as a random effect. Assessment will be made of the quadrature fit, with generalised estimating equations considered as an alternative if necessary.

For the clinical outcomes of changes at 9 months versus baseline in weight, haemoglobin, CD4 count, and lipids (total cholesterol, HDL cholesterol, LDL cholesterol, triglycerides), we will present the mean values and mean changes with standard errors, by group. We will compare between groups using linear regression, adjusted for the same variables as in the primary outcome analysis plus the baseline value of the respective outcome. The analysis of these outcomes will initially be restricted to participants who have the respective outcome data at 9 months. If there is a lot of missing outcome data, then we will also consider using multiple imputation for these outcomes, using baseline variables and measurements from the 3 and 6 month visits as appropriate (10). In addition, we will perform similar analyses for BMI, systolic and diastolic blood pressure, and total cholesterol/HDL cholesterol ratio. Of note, BMI and systolic and diastolic blood pressure will only be assessed among adults.

We will present the number and proportion of participants who had any new WHO 3 or 4 events, or died, by 9 months. We will describe the events. If data allow (i.e., enough events), we will compare between groups using Cox proportional hazards regression (with outcome of time to any new WHO 3

DocuSign Envelope ID: C2EAC1E7-7B2D-4962-BED6-57122FAF0196

or 4 events, or death), adjusted for the stratification factors. This analysis will include follow-up time to the last visit for each participant, with those not observed to have any such events censored at the minimum of the last visit or 130 weeks after randomisation.

We will present the number and proportion of participants who had any grade 3 or 4 AEs by 9 months, and summarise the overall numbers of AEs, grading, relationship to treatment, treatment changes, outcomes, event names. We will compare the number of participants with any grade 3 or 4 AEs between groups using logistic regression, adjusted for the stratification factors. This analysis will be done firstly among all participants (with the limitation that there may be unreported adverse events among participants who did not attended the final visit) and secondly restricted to participants who completed follow-up at 9 months. We will present and analyse SAEs similarly.

For the analyses of outcomes such as WHO events, deaths, AEs and SAEs which are described above as “by 9 months”, we will include any such events occurring up to the upper limit of the 9 month analysis window, namely 130 weeks (see section 3.7). If we are notified of any events occurring after this time, we will describe such events but they would not be included in the analyses.

We will present the number and proportion of participants with major drug resistance mutations at baseline (overall, and separately by randomised group; also reporting if any samples did not undergo successful RT-PCR amplification). We will present similar results for participants with unsuppressed viral load (>50 copies/ml) at 9 months, by randomised group.

6.3 Missing data

Where applicable, percentages will be of non-missing values, with the number (%) of missing values given if data are not complete. As detailed in section 6.2, main analyses of the primary outcome – and the virological secondary outcomes – will include all participants as randomised with missing data counted as failures. For the remaining secondary outcomes, section 6.2 details how missing data will be handled in each case.

6.4 Additional analyses

The long term follow up endpoint at 24 months will be assessed once all participants have passed that time point. All participants will be included as randomised, with those LTFU, died or changed regimen counted as failures like for the primary outcome analysis. Of note, dolutegravir (an integrase strand transfer inhibitor, INSTI) is being rolled out in Lesotho in 2020, and it is likely that a substantial proportion of our trial participants will be transitioned to dolutegravir before their 24 month follow up, potentially rendering the planned long term follow up analysis of limited clinical interest.

A separate analysis plan will be developed for the cost-effectiveness analyses (an exploratory outcome).

6.5 Harms

Safety data are included as secondary endpoints (see section 6.2).

For the laboratory parameters indicated in Table 3, the CTCAE categorisations will be applied as indicated in the table. We will present the number and proportion of participants with a measurement at each scheduled visit, by randomised group; and the numbers and proportions of

DocuSign Envelope ID: C2EAC1E7-7B2D-4962-BED6-57122FAF0196

participants with gradings 3 and 4 for each scheduled visit, by randomised group, of those with a measurement at the given visit. Of note, laboratory parameters graded 1-4 according to CTCAE would not have been reported as adverse events if they were determined by the clinical team to be not clinically significant. Laboratory abnormalities which occurred before study enrolment would be captured by the comorbidity question on the baseline form but would not otherwise be captured as an adverse event.

6.6 Statistical software

Analyses will be conducted in Stata version 15.

7. References

1. Gamble C, Krishan A, Stocken D, Lewis S, Juszczak E, Doré C, et al. Guidelines for the Content of Statistical Analysis Plans in Clinical Trials. JAMA. 2017 19;318(23):2337–43.

2. Amstutz A, Nsakala BL, Vanobberghen F, Muhairwe J, Glass TR, Achieng B, et al. SESOTHO trial (“Switch Either near Suppression Or THOusand”) – switch to second-line versus WHO-guided standard of care for unsuppressed patients on first-line ART with viremia below 1000 copies/mL: protocol of a multicenter, parallel-group, open-label, randomized clinical trial in Lesotho, Southern Africa. BMC Infect Dis [Internet]. 2018 [cited 2018 Feb 13];18. Available from: https://www.readcube.com/articles/10.1186/s12879-018-2979-y

3. Pocock S, et al. Subgroup analysis, covariate adjustment and baseline comparisons in clinical trial reporting: current practice and problems. Stat Med. 2002;21:2917–30.

4. The CONSORT Group. CONSORT Statement [Internet]. [cited 2012 Mar 22]. Available from: http://www.consort-statement.org/consort-statement/13-19---results/item15_baseline-data/

5. World Health Organization. Body mass index - BMI [Internet]. [cited 2017 Feb 23]. Available from: http://www.euro.who.int/en/health-topics/disease-prevention/nutrition/a-healthy-lifestyle/body-mass-index-bmi

6. US Department of Health and Human Sciences. Common Terminology Criteria for Adverse Events (CTCAE) [Internet]. 2009 [cited 2020 Mar 12]. Available from: https://www.eortc.be/services/doc/ctc/CTCAE_4.03_2010-06-14_QuickReference_5x7.pdf

7. Cockcroft D, Gault M. Prediction of creatinine clearance from serum creatinine. Nephron. 1976;16(1):31–41.

8. Stanford University. HIV drug resistance database [Internet]. [cited 2012 Dec 21]. Available from: http://hivdb.stanford.edu/

9. Norton EC, Miller MM, Kleinman LC. Computing Adjusted Risk Ratios and Risk Differences in Stata. Stata J Promot Commun Stat Stata. 2013 Sep;13(3):492–509.

10. Sullivan TR, White IR, Salter AB, Ryan P, Lee KJ. Should multiple imputation be the method of choice for handling missing data in randomized trials? Stat Methods Med Res. 2016 Dec 19;0962280216683570.

8. Shell tables

Presented below are the shell tables for the baseline characteristics, to illustrate the variables included. Further tables will be developed to present the analyses described above.

Table 8.1. Baseline characteristics by group: demographics.

	Control	Intervention	Total
Number randomised			
Child [1]			
Sex, female			
Age, years			
Means of transportation to health facility			
Taxi			
Walk			
Own car			
etc			
Travel time to health facility, minutes			
Cost to travel to health facility, LTL/ZAR			
Among adults			
Age, years			
Regular sex partner			
HIV status of current partner			
Don't know			
Positive and on ART			
Positive but don't know if on ART			
Positive but not on ART			
Recently tested negative			
No current partner			
Number of children			
0			
1			
2			
≥3			
Disclosure to current partner			
Education			
Did not complete primary			
Completed primary			
Completed secondary			
Completed tertiary			

DocuSign Envelope ID: C2EAC1E7-7B2D-4962-BED6-57122FAF0196

Employment			
Employed in Lesotho			
Employed in South Africa			
Self-employed with regular income			
No regular income			
Alcohol use [2]			
Illicit use of local drug (dacha)			
Among children			
Age, years			
Care giver situation			
Mother			
Family member			
etc			
Orphan status			
No			
Yes, single			
Yes, double			

Results are n (% of those with non-missing data) for categorical variables and median (IQR) [range] for continuous variables.
[1] Defined as <16 years old.
[2] Female: at least 2 drinks every day or regularly 4 or more drinks on one occasion; Male: at least 3 drinks every day or regularly 5 or more drinks on one occasion.

DocuSign Envelope ID: C2EAC1E7-7B2D-4962-BED6-57122FAF0196

Table 8.2. Baseline characteristics by group: clinical.

	**Control**	**Intervention**	**Total**
Number randomised			
ART history; exposure to:			
TDF			
ABC			
AZT			
3TC			
d4T			
EFV			
NVP			
Current ART regimen at screening			
TDF/3TC/EFV			
ABC/3TC/EFV			
AZT/3TC/EFV			
TDC/3TC/NVP			
AZT/3TC/NVP			
Clinical WHO stage			
1			
2			
History of TB			
Isoniazid Preventive Therapy (IPT) given today			
Co-trimoxazole given today			
Other concomitant treatment			
Among adults			
Weight, kg			
BMI, kg/m^2			
Blood Pressure, systolic, mmHg			
Blood Pressure, diastolic, mmHg			
Among children			
Weight, kg			

Results are n (% of those with non-missing data) for categorical variables and median (IQR) [range] for continuous variables.

DocuSign Envelope ID: C2EAC1E7-7B2D-4962-BED6-57122FAF0196

Table 8.3. Baseline characteristics by group: laboratory.

	**Control**	**Intervention**	**Total**
Number randomised			
VL, copies/ml			
CD4 count, cells/mm^3			
Haemoglobin, g/dL			
Creatinine, micromol/L			
eGFR, ml/min/1.73m^2 [1]			
ALT, U/L			
AST, U/L			
Total cholesterol, mmol/L			
HDL, mmol/L			
LDL, mmol/L			
Triglycerides, mmol/L			

Results are median (IQR) [range].
[1] Estimated using Cockcroft-Gault equation (7).
