# Supplementary material for: Switch to second-line versus continued first-line antiretroviral therapy for patients with low-level HIV-1 viremia: An open-label randomized controlled trial in Lesotho
Source: PLoS Med. 2020 Sep 16;17(9):e1003325. doi: 10.1371/journal.pmed.1003325 (PMC7494118; doi:10.1371/journal.pmed.1003325)
Supplement: S1 Table — (DOCX) [file pmed.1003325.s004.docx]

**S1 Table: Enrolment by site**

|  | **Control group (n=40)** | **Switch group (n=40)** | **Total (n=80)** |
| --- | --- | --- | --- |
| Butha-Buthe Government Hospital | 18 (45%) | 17 (43%) | 35 (44%) |
| Seboche Mission Hospital | 7 (18%) | 6 (15%) | 13 (16%) |
| St Paul Health Centre | 0 | 1 (3%) | 1 (1%) |
| St Peters Health Centre | 0 | 1 (3%) | 1 (1%) |
| Muela Health Centre | 1 (3%) | 2 (5%) | 3 (4%) |
| Mokhotlong Hospital | 1 (3%) | 2 (5%) | 3 (4%) |
| Senkatana Hospital | 9 (23%) | 6 (15%) | 15 (19%) |
| Motebang Hospital | 4 (10%) | 5 (13%) | 9 (11%) |
