# Supplementary material for: Switch to second-line versus continued first-line antiretroviral therapy for patients with low-level HIV-1 viremia: An open-label randomized controlled trial in Lesotho
Source: PLoS Med. 2020 Sep 16;17(9):e1003325. doi: 10.1371/journal.pmed.1003325 (PMC7494118; doi:10.1371/journal.pmed.1003325)
Supplement: S2 Table — (DOCX) [file pmed.1003325.s005.docx]

**S2 Table: Sensitivity analyses for the primary endpoint**

|  | **VL <50 copies/mL at 36 weeks** | | | **Odds ratio (95% CI) [1]** | **Risk diff (95% CI) [1,2]** | **P-value [1]** |
| --- | --- | --- | --- | --- | --- | --- |
|  | **Overall (n=80)** | **Control group (n=40)** | **Switch group (n=40)** |  |  |  |
| Primary analysis [3] | 32/80 (40%) | 10/40 (25%) | 22/40 (55%) | 3·55 (1·37,9·24) | 29% (8,50) | 0·009 |
| Sensitivity: unadjusted | 32/80 (40%) | 10/40 (25%) | 22/40 (55%) | 3·67 (1·42,9·47) | 30% (10,50) | 0·007 |
| Sensitivity: model adjusted for some baseline covariates with imbalance [4] | 32/80 (40%) | 10/40 (25%) | 22/40 (55%) | 3·40 (1·19,9·74) | 25% (4,46) | 0·02 |
| Sensitivity: model adjusted for all baseline covariates with imbalance [5] | 32/79 (41%) | 10/39 (26%) | 22/40 (55%) | 5·59 (1·58,19·8) | 31% (10,51) | 0·007 |
| Sensitivity: as above plus adjusted for time on ART [6] | 32/79 (41%) | 10/39 (26%) | 22/40 (55%) | 5·57 (1·58,19·7) | 30% (10,51) | 0·008 |
| Sensitivity: per protocol set [7] | 30/73 (41%) | 8/36 (22%) | 22/37 (59%) | 5·15 (1·81,14·7) | 37% (16,58) | 0·002 |
| Sensitivity: VLs in pre-defined window 32-40 weeks [8,9] | 28/62 (45%) | 8/32 (25%) | 20/30 (67%) | 6·13 (2·01,18·7) | 42% (19,65) | 0·001 |
| Sensitivity: VLs in wider pre-defined window 32-44 weeks [10] | 32/74 (43%) | 10/38 (26%) | 22/36 (61%) | 4·28 (1·59,11·5) | 35% (13,56) | 0·004 |
| Sensitivity: only including those with baseline VL≥200 copies/mL [11] | 19/55 (35%) | 5/29 (17%) | 14/26 (54%) | 5·31 (1·51,18·7) | 34 (10,58) | 0·009 |

Abbreviations: ART (antiretroviral therapy), CI (confidence interval), VL (viral load)

[1] Switch versus control group, estimated by logistic regression

[2] Confidence intervals estimated using delta method

[3] Adjusted for demographic group and baseline VL. Outcome analysis window 32-52 weeks after enrolment.

[4] Adjusted for demographic group, baseline VL, alcohol use, co-trimoxazole use, and other concomitant medications, which were determined a priori to be potentially clinically important confounders.

[5] Adjusted for variables as in footnote 4, plus means of transportation to the health facility, number of children, and education. One adult in the control group with missing number of children omitted.

[6] Post hoc analysis: as footnote 5 but also adjusted for time on ART at baseline

[7] Including only participants that finished the 36 weeks according to the protocol (=alive, retained in care, no change in regimen line other than that indicated by the randomisation (regardless of the reason), VL measurement available at 36 weeks). Omits 3 participants in control group who changed regimen line and 1 participant in control group who did not have VL measured, and 3 participants in switch group who did not have VL measured (one of whom had anyway switched back to first line)

[8] Including only VLs measured within the pre-defined windows

[9] Adjusted for baseline VL only since all three of the children included achieved VL <50 copies/mL at 36 weeks.

[10] As footnote 8 but using 30·4 days per month for the upper limit of 10 months (32-44 weeks).

[11] Post hoc analysis, motivated by guidelines using threshold of 200 copies/mL
