# Supplementary material for: Switch to second-line versus continued first-line antiretroviral therapy for patients with low-level HIV-1 viremia: An open-label randomized controlled trial in Lesotho
Source: PLoS Med. 2020 Sep 16;17(9):e1003325. doi: 10.1371/journal.pmed.1003325 (PMC7494118; doi:10.1371/journal.pmed.1003325)
Supplement: S3 Table — (DOCX) [file pmed.1003325.s006.docx]

**S3 Table: Socio-demographic characteristics of trial participants**

|  | **Control group (n=40)** | **Switch group (n=40)** | **Total (n=80)** |
| --- | --- | --- | --- |
| **Among all participants** |  |  |  |
| Means of transportation to health facility |  |  |  |
| Taxi | 28 (70%) | 23 (58%) | 51 (64%) |
| Walk | 10 (25%) | 15 (38%) | 25 (31%) |
| Own car | 2 (5%) | 2 (5%) | 4 (5%) |
| Travel time to health facility, minutes [1] | 60 (30-80) [15-180] | 30 (25-60) [10-120] | 40 (30-60) [10-180] |
| Cost to travel to health facility, LSL/ZAR | 16 (6-20) [0-80] | 9 (0-16) [0-65] | 10 (0-18) [0-80] |
| **Among adults only** | **N=37** | **N=39** | **N=76** |
| Regular sex partner | 27 (73%) | 26 (67%) | 53 (70%) |
| HIV status of current partner |  |  |  |
| Don’t know | 6 (16%) | 4 (10%) | 10 (13%) |
| Positive and on ART | 14 (38%) | 13 (33%) | 27 (36%) |
| Positive but don’t know if on ART | 2 (5%) | 1 (3%) | 3 (4%) |
| Positive but not on ART | 2 (5%) | 2 (5%) | 4 (5%) |
| Recently tested negative | 4 (11%) | 7 (18%) | 11 (14%) |
| No current partner | 9 (24%) | 12 (31%) | 21 (28%) |
| Number of children [1] |  |  |  |
| 0 | 3 (8%) | 10 (26%) | 13 (17%) |
| 1 | 5 (14%) | 6 (15%) | 11 (15%) |
| 2 | 11 (31%) | 8 (21%) | 19 (25%) |
| ≥3 | 17 (47%) | 15 (38%) | 32 (43%) |
| Disclosure to current partner | 35 (95%) | 37 (95%) | 72 (95%) |
| Education |  |  |  |
| Did not complete primary | 10 (27%) | 9 (23%) | 19 (25%) |
| Completed primary | 12 (32%) | 18 (46%) | 30 (39%) |
| Completed secondary | 10 (27%) | 11 (28%) | 21 (28%) |
| Completed tertiary | 5 (14%) | 1 (3%) | 6 (8%) |
| Employment |  |  |  |
| Employed in Lesotho | 10 (27%) | 7 (18%) | 17 (22%) |
| Employed in South Africa | 1 (3%) | 2 (5%) | 3 (4%) |
| Self-employed with regular income | 5 (14%) | 9 (23%) | 14 (18%) |
| No regular income | 21 (57%) | 21 (54%) | 42 (55%) |
| **Among children (<16 years) only** | **N=3** | **N=1** | **N=4** |
| Care giver situation |  |  |  |
| Mother | 0 | 1 | 1 |
| Family member | 3 | 0 | 3 |
| Orphan status |  |  |  |
| No | 1 | 1 | 2 |
| Yes, single | 2 | 0 | 2 |

Results are number (% of those with non-missing data) for categorical variables and median (IQR) [range] for continuous variables

Abbreviations: ART (antiretroviral therapy), IQR (interquartile range), LSL (Lesotho Loti), ZAR (South African Rand),

[1] Missing for one participant in control group
