# Supplementary material for: Switch to second-line versus continued first-line antiretroviral therapy for patients with low-level HIV-1 viremia: An open-label randomized controlled trial in Lesotho
Source: PLoS Med. 2020 Sep 16;17(9):e1003325. doi: 10.1371/journal.pmed.1003325 (PMC7494118; doi:10.1371/journal.pmed.1003325)
Supplement: S4 Table — (DOCX) [file pmed.1003325.s007.docx]

**S4 Table: Baseline genotypic resistance testing**

|  | **Control group (n=40)** | **Switch group (n=40)** | **Total (n=80)** |
| --- | --- | --- | --- |
| Number of participants with sample available for sequencing | 37 (93%) | 37 (93%) | 74 (93%) |
| Number of samples successfully sequenced | 14 (35%) | 15 (38%) | 29 (36%) |
| **Of those successfully sequenced** | **N=14** | **N=15** | **N=29** |
| Subtype C | 14 (100%) | 15 (100%) | 29 (100%) |
| Any mutation [1] | 11 (79%) | 14 (93%) | 25 (86%) |
| Stanford level 5 resistance to an NRTI | 11 (79%) | 14 (93%) | 25 (86%) |
| Stanford level 5 resistance to an NNRTI | 11 (79%) | 14 (93%) | 25 (86%) |
| Stanford level 2 resistance to a PI (M41I mutation; none higher resistance) | 1 (7%) | 0 | 1 (3%) |
| ARVs on at enrolment: Stanford level 5 resistance to: |  |  |  |
| At least two | 11 (79%) | 14 (93%) | 25 (86%) |
| All three | 9 (64%) | 7 (47%) | 16 (55%) |
| ARVs switched to following randomisation: Stanford level 4-5 resistance to: [2] |  |  |  |
| At least one | NA | 14 (93%) | NA |
| At least two | NA | 2 (13%) | NA |

Abbreviations: ARV (antiretroviral), LPV/r (lopinavir/ritonavir), NRTI (nucleoside reverse transcriptase inhibitor), NNRTI (non-nucleoside reverse transcriptase inhibitor), PI (protease inhibitor)

[1] Resistance was assessed using the Stanford algorithm ([www.hivdb.stanford.edu](http://www.hivdb.stanford.edu), accessed 25 April 2020) and considering variants present at ≥10%. Stanford levels are 1-5 as follows: 1=susceptible, 2=potential low-level resistance, 3=low-level resistance, 4=intermediate resistance, 5=high-level resistance.

[2] Includes all ARVs in new regimen, even if was present in regimen at screening/enrolment
