# Supplementary material for: Switch to second-line versus continued first-line antiretroviral therapy for patients with low-level HIV-1 viremia: An open-label randomized controlled trial in Lesotho
Source: PLoS Med. 2020 Sep 16;17(9):e1003325. doi: 10.1371/journal.pmed.1003325 (PMC7494118; doi:10.1371/journal.pmed.1003325)
Supplement: S5 Table — ART, antiretroviral therapy. (DOCX) [file pmed.1003325.s008.docx]

**S5 Table: ART regimens over time**

|  | **Control group (n=40)** | **Switch group (n=40)** |
| --- | --- | --- |
| **ART regimen provided at:** |  |  |
| *Week 0* |  |  |
| TDF/3TC/EFV | 23 (57%) | 0 (0%) |
| ABC/3TC/EFV | 3 (8%) | 0 (0%) |
| AZT/3TC/EFV | 6 (15%) | 0 (0%) |
| TDF/3TC/NVP | 4 (10%) | 0 (0%) |
| AZT/3TC/NVP | 4 (10%) | 0 (0%) |
| TDF/3TC/LPV/r | 0 (0%) | 6 (15%) |
| ABC/3TC/LPV/r | 0 (0%) | 4 (10%) |
| AZT/3TC/LPV/r | 0 (0%) | 30 (75%) |
| TDF/3TC/DTG | 0 (0%) | 0 (0%) |
| ABC/3TC/DTG | 0 (0%) | 0 (0%) |
| *Week 12* |  |  |
| TDF/3TC/EFV | 22 (55%) | 1 (3%) |
| ABC/3TC/EFV | 3 (8%) | 0 (0%) |
| AZT/3TC/EFV | 6 (15%) | 0 (0%) |
| TDF/3TC/NVP | 4 (10%) | 0 (0%) |
| AZT/3TC/NVP | 4 (10%) | 0 (0%) |
| TDF/3TC/LPV/r | 0 (0%) | 6 (15%) |
| ABC/3TC/LPV/r | 0 (0%) | 4 (10%) |
| AZT/3TC/LPV/r | 1 (3%) | 28 (72%) |
| TDF/3TC/DTG | 0 (0%) | 0 (0%) |
| ABC/3TC/DTG | 0 (0%) | 0 (0%) |
| *Week 24* |  |  |
| TDF/3TC/EFV | 22 (55%) | 1 (3%) |
| ABC/3TC/EFV | 2 (5%) | 0 (0%) |
| AZT/3TC/EFV | 6 (15%) | 0 (0%) |
| TDF/3TC/NVP | 4 (10%) | 0 (0%) |
| AZT/3TC/NVP | 4 (10%) | 0 (0%) |
| TDF/3TC/LPV/r | 0 (0%) | 6 (15%) |
| ABC/3TC/LPV/r | 0 (0%) | 4 (10%) |
| AZT/3TC/LPV/r | 1 (3%) | 28 (72%) |
| TDF/3TC/DTG | 0 (0%) | 0 (0%) |
| ABC/3TC/DTG | 1 (3%) | 0 (0%) |
| *Week 36* |  |  |
| TDF/3TC/EFV | 22 (56%) | 1 (3%) |
| ABC/3TC/EFV | 2 (5%) | 0 (0%) |
| AZT/3TC/EFV | 5 (13%) | 0 (0%) |
| TDF/3TC/NVP | 3 (8%) | 0 (0%) |
| AZT/3TC/NVP | 4 (10%) | 0 (0%) |
| TDF/3TC/LPV/r | 0 (0%) | 6 (15%) |
| ABC/3TC/LPV/r | 0 (0%) | 4 (10%) |
| AZT/3TC/LPV/r | 1 (3%) | 28 (72%) |
| TDF/3TC/DTG | 1 (3%) | 0 (0%) |
| ABC/3TC/DTG | 1 (3%) | 0 (0%) |

Abbreviations: ABC (abacavir), ART (antiretroviral therapy), AZT (zidovudine), DTG (dolutegravir), EFV (efavirenz), LPV/r (lopinavir/ritonavir), NVP (nevirapine), TDF (tenofovir), 3TC (lamivudine)
