# Supplementary material for: Switch to second-line versus continued first-line antiretroviral therapy for patients with low-level HIV-1 viremia: An open-label randomized controlled trial in Lesotho
Source: PLoS Med. 2020 Sep 16;17(9):e1003325. doi: 10.1371/journal.pmed.1003325 (PMC7494118; doi:10.1371/journal.pmed.1003325)
Supplement: S6 Table — (DOCX) [file pmed.1003325.s009.docx]

**S6 Table: Adverse Events – summary**

|  | **Control group (n=40)** | **Switch group (n=40)** | **Total**  **(n=80)** |
| --- | --- | --- | --- |
| Participants with at least one AE [1] | 21 (53%) | 34 (85%) | 55 (69%) |
| Participant with at least one grade 3/4 AE | 1 (3%) | 1 (3%) | 2 (3%) |
| Number of AEs per participant, median (IQR) [range] | 1 (0-1) [0-6] | 1 (1-2) [0-6] | 1 (0-2) [0-6] |
| **Total number AEs** | **N=42** | **N=60** | **N=102** |
| CTCAE grade |  |  |  |
| 1 | 36 (86%) | 52 (87%) | 88 (86%) |
| 2 | 5 (12%) | 7 (12%) | 12 (12%) |
| 3 | 1 (2%) | 1 (2%) | 2 (2%) |
| 4 | 0 | 0 | 0 |
| Relationship to treatment |  |  |  |
| Not related | 33 (79%) | 28 (47%) | 61 (60%) |
| May be related | 8 (19%) | 30 (50%) | 38 (37%) |
| Related | 1 (2%) | 2 (3%) | 3 (3%) |
| Outcome |  |  |  |
| Resolved, no sequelae | 40 (95%) | 58 (97%) | 98 (96%) |
| Ongoing | 2 (5%) | 0 | 2 (2%) |
| Unknown | 0 | 2 (3%) | 2 (2%) |

Abbreviations: AE (adverse event), CI (confidence interval), CTCAE (common terminology criteria for adverse events) IQR (interquartile range), OR (odds ratio), VL (viral load)

[1] Logistic regression model comparing the two groups and adjusted for demographic group and baseline VL: OR=5·66 (95% CI 1·90,16·9), risk difference=34% (15,53), p=0·002. Restricted to the 78 participants who completed 36 weeks follow up: OR=5·76 (1·92,17·3), risk difference=35% (15,54), p=0·002.
