# Supplementary material for: Switch to second-line versus continued first-line antiretroviral therapy for patients with low-level HIV-1 viremia: An open-label randomized controlled trial in Lesotho
Source: PLoS Med. 2020 Sep 16;17(9):e1003325. doi: 10.1371/journal.pmed.1003325 (PMC7494118; doi:10.1371/journal.pmed.1003325)
Supplement: S7 Table — (DOCX) [file pmed.1003325.s010.docx]

**S7 Table: Adverse Events – list**

|  | **Control group** | **Switch group** | **Total** |
| --- | --- | --- | --- |
| **Total** | **42** | **60** | **102** |
| Gastrointestinal (diarrhoea, vomiting, nausea, abdominal pain, dizziness) | 1 | 17 | 18 |
| Peripheral neuropathy | 0 | 3 | 3 |
| Drop of serum creatinine clearance <50mL/min | 0 | 1 | 1 |
| Transient ALT/AST elevation | 0 | 3 | 3 |
| Anaemia/drop in haemoglobin | 1 | 1 | 2 |
| Acute respiratory infection | 12 | 5 | 17 |
| Headache | 6 | 1 | 7 |
| Musculoskeletal pain | 5 | 5 | 10 |
| Rash | 3 | 7 | 10 |
| Genital infection | 2 | 1 | 3 |
| Aphthous ulcer | 1 | 3 | 4 |
| Conjunctivitis | 2 | 1 | 3 |
| Other unrelated AEs | 9 | 12 | 21 |

Abbreviations: AE (adverse events), ALT (alanine aminotransferase), AST (aspartate aminotransferase)
