# Supplementary material for: Switch to second-line versus continued first-line antiretroviral therapy for patients with low-level HIV-1 viremia: An open-label randomized controlled trial in Lesotho
Source: PLoS Med. 2020 Sep 16;17(9):e1003325. doi: 10.1371/journal.pmed.1003325 (PMC7494118; doi:10.1371/journal.pmed.1003325)
Supplement: Attachment — Submitted filename: SESOTHO_PLoSMed_response_letter_10.07.20_final.docx [file pmed.1003325.s011.docx]

**Response to Editors and Reviewers**

***** Requests from the editors:**

To your data statement, please add non-author contact details for those wishing to inquire about access to the full dataset.

***Response:*** *We have added a non-author email address as requested and adapted our data-statement as follows:*

Data were accessible to all authors after data freezing on 23.05.2020. A subset of the key pseudo-anonymised individual participant data collected during the study, along with a data dictionary, will be made available at the time of publication through the data repository Zenodo. The full data-set will be made available upon request to the Clinical Statistics and Data Management Group of the Department of Medicine at the Swiss Tropical and Public Health Institute ([clin.stats.dm@swisstph.ch](mailto:clin.stats.dm@swisstph.ch)) and after signing a data confidentiality agreement.

Please quote the setting in your title, and substitute a colon before the study descriptor. 
***Response****: We adapted accordingly.*

We ask you to add a sentence to the abstract to quote the higher proportion of "switch" participants experiencing adverse events (as discussed around line 356). 
***Response****: We added the following sentence to the abstract, section “Methods and Findings”:*

34 (85%) participants in switch group and 21 (53%) in control group experienced at least one adverse event (p=0.002).

Please add a new final sentence to the "methods and findings" subsection of your abstract, beginning "Study limitations include ..." or similar and quoting 2-3 of the study's main limitations. 
***Response****: We added the following sentence:*

Study limitations include a follow-up period too short to observe differences in clinical outcomes, missing values in CD4 cell counts due to national stockout of reagents during the study and limited generalizability of findings to other than NNRTI-based first-line ART regimens.

Please add "In this study ..." or similar at line 65. 
***Response****: We modified accordingly*

At line 284 you use the word "trend". Please make it clear whether this refers to a statistical trend (the relevant p value appears to be 0.2), and we suggest adapting the wording to the form "X was non-significantly greater than Y" if appropriate. 
***Response****: We adjusted as follows:*

The intervention effect among those with baseline VL 600-999 copies/mL (9/13 [69%] in the switch and 2/11 [18%] in the control group; adjusted risk difference 48% [95% CI 21 to 76]) was non-significantly greater than the effect among those with baseline VL 100-599 copies/mL (12/27 [48%] in the switch and 8/29 [28%] in the control group; adjusted risk difference 18% (95% CI -5 to 42); interaction p=0.20).

Please revisit the first paragraph of your discussion section, which should summarize the study's findings. Rather than "... we have demonstrated ... increases viral suppression", we ask you to report the study's findings in the paste tense (e.g., "... we observed that ... increased ...").
***Response****: We adapted the first paragraph of the discussion accordingly.*

Where you make a claim of "the first", as at line 364 for example, please add "to our knowledge" or similar. 
***Response****: We adapted as follows:*

To our knowledge, the SESOTHO trial is the first randomized controlled trial that addresses ART switch in persons with HIV-1 low-level viremia. We observed that switching from NNRTI-based first-line to PI-based second-line ART among patients with persistent LLV (between 100-999 copies/mL) increased viral suppression (<50 copies/mL) at 36 weeks compared to continued first-line ART.

Please move reference call-outs before punctuation throughout the paper, e.g. "... onward transmission [2,3].".
***Response****: We adapted accordingly.*

Please remove trademarks from the paper. 
***Response****: We adapted accordingly.*

Please remove the information on data sharing, funding and conflict of interest from the end of the text. This information will appear in the metadata in the event of publication, via information provided in the submission form. 
***Response****: We adapted accordingly.*

Please revisit your reference list to ensure that all citations meet journal format. All italics should be converted into plain text; six author names should be listed rather than 3, followed by "et al." where appropriate; and for reference 3, for example, "Lancet" will suffice for the journal name. 
***Response****: We adapted accordingly.*

Please rename figure 1 "Participant flow diagram" or similar. 
***Response****: We adapted accordingly.*

We ask that you attach a completed CONSORT checklist for your study, referred to in the methods section ("see S1_CONSORT_Checklist" or similar). In the checklist, please refer to individual items by section (e.g., "Methods") and paragraph numbers rather than by page or line numbers, as the latter generally change in the event of publication. 
***Response****: We attached an updated CONSORT checklist.*

**Comments from the reviewers:
*** Reviewer #1:** 
In this trial, participants with a VL >100 were switched, or not, and 36 weeks later their chances of being <50 was assessed.
The failure threshold of <1000 was chosen based on transmission risk. The authors need to explain the negative clinical implications of low level viraemia beyond simply having a greater risk of viral failure. People who were switched earlier experienced more toxicity and worse adherence, so the clinical benefits need to be made clear.  The fact that low level viraemia is a risk factor for viral failure is logical, just as being aged 49 is a "risk factor" for turning 50. 
***Response****: Thank you for this important point. We revised section “Introduction” by adding:*

Yet, several large cohort studies from resource-rich and resource-limited settings observed a higher risk of developing major drug resistance mutations [8,9], virologic failure [3–8], AIDS-defining events and mortality [9,10] among patients with sustained detectable VL but below 1000 copies/mL as compared to patients with undetectable VL.

*and in section “Discussion” we added another important point:*

Moreover, in resource-limited settings with less frequent virologic monitoring and high rates of loss to follow-up, one may argue that the use of the lowest possible sensitive treatment failure threshold is important to be able to react in time, compared to resource-rich settings with frequent and reliable laboratory monitoring.

Why was 36 weeks chosen for time to suppression?
***Response****: We added the following rationale in the Methods section:*

The rationale for this time-point was that, according to WHO and Lesotho national guidelines, VL is measured 6 months after switch to second-line ART. If this VL is above 1000 copies/mL, enhanced adherence counselling is provided and a follow-up VL performed 3 months later. Thus, 36 weeks was the earliest possible time point to meet the criteria of virologic failure (versus viral resuppression) after switch to second-line ART.

Line 101: "Viral suppression should be achieved by latest six months after ART initiation". This depends on viral load at initiation and regimen.
***Response****: We agree and therefore deleted this sentence in section “Introduction”.*

Line 192: What stats programme was used?
***Response****: We have added the following information in the section “Methods”:*

Analyses were performed using Stata (StataCorp. 2017. *Version 15*).

Line 215: Why was self-report of no missed doses in the previous four weeks chosen to define "good adherence"
***Response****: In daily practice and in previous studies conducted in Lesotho we observed that the pill-count was not such a reliable adherence measure, particularly when it comes to multi-pill regimens (as is the case for those who were switched to second-line). We thus decided to go for a simple but validated self-reported adherence assessment. The simple question if one or more doses were missed during the last 4 weeks was validated as a good proxy for adherence problems in different settings (Glass et al. JAIDS, 2015 & Paton et al. EARNEST trial, NEJM, 2014). We added these two references in section “Statistical methods”, line 229.*

Line 223: It would be helpful if the viral load stratifications in the baseline characteristics and outcomes were matched for comparability. As currently presented, baseline characteristics are 100-599 and 600-999, whereas outcomes are 20,100,200,400, and 1000.
***Response****: We have added this analysis to the paper.*

Line 397: "In this current study, we found higher proportions of viral suppression in the
switch group when considering virologic failure thresholds other than <50 copies/mL, i.e. 20,
100, 200, and 400 copies/mL, but not for the current WHO recommended threshold of 1000
400 copies/mL."
- Is this simply because of time to suppression?
***Response****: In the switch group we observed a higher proportion of participants who had suppressed VL (at 36 weeks) below 20, 50, 100, 200 and 400 copies/mL than in the control group. However, when considering a VL of <1000 copies/mL at 36 weeks, the proportions were similar, at 80% in control group and 85% in intervention group. In other words, a progress from LLV to viral rebound above 1000 copies/mL was relatively rare in both groups. This may be due to the relatively short follow-up period. We clarified this in the section “Discussion” as follows:*

In this current study, we found higher proportions of viral suppression in the switch group when considering virologic outcomes at 36 weeks other than <50 copies/mL, i.e. 20, 100, 200, 400, and 600 copies/mL, but not for the current WHO recommended threshold of 1000 copies/mL. Worsening of LLV in terms of a viral rebound above 1000 copies/mL at 36 weeks was rare (17%) and similar in both groups.

Line 446: "individual benefits need to be balanced against the public health implications.". 
- Individual benefits also need to be balanced against the individual harm of a higher frequency of side effects and worse adherence (which did not lead to observable worse outcomes in this trial but could lead to drug resistance later on)
***Response****: We agree with the reviewer that virologic outcomes need to be balanced against clinical outcomes. We adapted line 464 and line 504 in section “Discussion”:*

Overall, these findings endorse a lower VL threshold in future WHO guidelines. However, higher rates of viral suppression should be balanced against higher rates of reported adverse-events, particularly gastro-intestinal side-effects due to lopinavir/ritonavir and their potential long-term consequences to adherence.

***** Reviewer #2:**

Review of ‘Switch to second-line versus continued first-line antiretroviral therapy for patients with low-level HIV-1 viremia – an open-label randomized controlled trial’

The manuscript describes the results of an open-label, randomized clinical trial to compare viral suppression (<50c/mL) at 36 weeks among patients with sustained low-level HIV-1 viremia on first-line NNRTI ART to second-line ART in Lesotho. The results of this trial are intended to inform WHO policy guidelines on the viral load threshold trigger for switching to second-line ART. The manuscript addresses an important question and is of interest to the general PLOS Medicine readership.

The manuscript is clearly written and the trial thoughtfully designed and executed. The statistical methodology is appropriate and well-described in the Methods section, with a few suggested additions/clarifications listed below. The introduction and discussion provide a good review of the literature to place the findings in context.

The trial had 90% power to detect a difference of 35% in the two-sided comparison of rates with n=40 per group. These calculations did not account for potential missing data if some participants were lost to follow up before their primary endpoint was measured however missing data wasn’t much of an issue (2% in switch group, 7% in control group). Results of the sensitivity analyses confirmed the primary analyses with only a slight attenuation of the effect size seen under one scenario and larger effect sizes seen in others. Post-hoc analyses were described as such.

The authors acknowledged a limitation of the primary endpoint of viral suppression at 36 weeks as a measure of treatment success rather than more clinically meaningful events or virologic rebound. Few of the latter were observed due to the relatively short follow-up period. In addition, there were very little data on immunologic outcomes due to stockout of CD4 reagents. The authors also acknowledge that the global introduction of dolutegravir-containing ART is likely to change the prevalence of drug-resistant treatment failure. These limitations raise some questions about the relevance of the current paper however, the authors are upfront about these limitations. The switch group experienced a significantly higher rate of (>1) AEs, mainly gastrointestinal, though not a higher rate of grade 3/4 AEs. Another limitation is that adherence was measured solely by self-report and not by other methods such as pill counts or electronic monitoring devices.

*Response: Thank you for your positive feedback and the comprehensive summary.*

Specific Comments:

- Please state the following in the Statistical Methods section: the software including version number used for statistical analyses, alpha level, whether 2-sided tests were used, any adjustment for multiple comparisons, handling of missing data. Was a multiplicity adjustment considered in the analyses of the 5 different VL thresholds?

***Response****:*

- *We have added the statistical software (see response to comment of reviewer 1).*
- *We prefer to avoid any statement about the level of significance for tests (i.e. alpha values and two-sided tests) and instead have added that estimates are reported with 95% confidence intervals.*
- *As per the statistical analysis plan: “No adjustments will be made for multiple testing; interpretations will be based on the strength of evidence of effect size and consistency of results for related outcomes” and we have now added this detail to the manuscript under “Statistical Methods” where we state:*

No adjustments were made for multiple testing.

- *As stated in the manuscript and in line with our statistical analysis plan: “Participants who were switched to next-line ART due to clinical failure prior to 36 weeks or did not have VL measured at 36 weeks were considered to have unsuppressed VL”. That is, participants with missing VL results at week 36 were treated as having unsuppressed VL for the primary outcome. For clinical, adherence and laboratory outcomes, however, analyses were performed among those with non-missing data; we have added this information to the manuscript under “Statistical Methods”:*

Clinical, adherence and laboratory outcomes were assessed among those with non-missing data.

*(please also further see response to your bullet 3)*

- Please rephrase the following text on line 284 so that an interaction p-value of 0.20 for a post-hoc analysis is not described as a “trend” towards a stronger intervention effect. “There was a trend towards a stronger intervention effect among those with baseline VL 600-999 copies/mL (9/13 [69%] in the switch and 2/11 [18%] in the control group; adjusted risk difference 48% [95% CI 21 to 76]) versus VL 100-599 copies/mL (12/27 [48%] in the switch and 8/29 [28%] in the control group; adjusted risk difference 18% (95% CI -5 to 42); interaction p=0.20).

***Response****: We adapted the sentence accordingly*

pg 13, line 297: Add “self-reported” in describing good adherence

***Response****: We adapted accordingly.*

- pg 15, Table 3: How was missing data handled in the linear regressions for the last 7 rows of the table where there was substantial missing data? Was multiple imputation considered? If the results are based on complete case analysis, the results on pg 14 should note that the significant findings should be interpreted with caution given the high level of missing data.

***Response****: As indicated in response to the first bullet point above, clinical outcomes were assessed among those with non-missing data only, and we apologize that this was not clear before. We agree that results of CD4 cell counts and lipid panels should be interpreted with caution due to the high amount of missingness and thus reworded as follows:*

There was no evidence of a difference in changes in mean body weight, haemoglobin, CD4 count and blood lipid levels between baseline and 36 weeks, apart from a slightly larger increase in triglycerides and total/HDL cholesterol ratio in the switch versus control groups, although these results should be interpreted with caution due to the large amount of missing data (table 3).

We further mention this as a limitation under “Discussion”:

Thirdly, due to a national stockout of CD4 reagents during the study period, data on immunological outcomes are too incomplete to draw any conclusions.

- Pg 16, line 358: Report p-value for comparison of related/maybe related AEs between groups

***Response****: We have added the following information in the results section:*

Overall, 41 (40%) AEs were judged as may be related (n=32) or related (n=9) to treatment, with more in the switch versus control group (27/40 [68%] versus 8/40 [20%], p<0.001).

- Pg 16, line 365: Change “switch” to “switching”

***Response****: We adapted accordingly.*

- Pg 16, line 366: Add “at 36 weeks” before “compared”

***Response****: We adapted accordingly.*

- Self-reported adherence was worse in switch group than control group (51% vs 72%, p=0.08) yet still saw a significant difference in the proportion with VL<50c/mL at 36 and 24 weeks in the switch group compared to the control group. Can the authors comment on how adherence might be improved?

***Response****: Worse self-reported adherence in the switch group may have been caused by the higher pill burden of the lopinavir/ritonavir-based second-line regimens that consisted of 5-6 pills per day or by the higher occurrence of adverse events. We clarified on the possible reasons for worse adherence in the switch as follows in the “Discussion” section:*

Self-reported drug-adherence was lower in the switch than control group, with only 51% reporting good adherence throughout follow-up in the switch compared to 72% in the control group. This may have been caused by the higher pill burden of the lopinavir/ritonavir-based second-line regimens that consisted of 5-6 pills per day or by the higher occurrence of AEs.

*We feel that further exploration of the adherence topic would go beyond the scope of this manuscript.*

***** Reviewer #3:** 

General comments
This is a highly relevant study which has been rigorously conducted in a setting representative of those were a majority of people living with HIV receive care. It addresses a common clinical problem in a randomized control trial. Although the occurrence of LLV during 1st line ART may decrease if dolutegravir replaces efavirenz as anchor drugs in sub-Saharan Africa, the findings of this study will still be of relevance, not least by highlighting and adding further evidence to the fact that attention must be paid to persons with detectable VL <1000 copies/ml.   
Major comments
Overall, I think the study is well performed and presented. I have no major comments on the paper. 
***Response:*** *Thank you very much for your positive feedback.*

Minor comments 
1.	In order to provide an estimate of the prevalence of LLV (according to the study definitions) in the uptake population, it would be interesting to present data from the total number of persons in the source cohort. In particular, it would be interesting to know how many had a VL in the range 100-999 copies/ml which was followed by VL<100 copies/ml (single measurement LLV). In the Hermans study from South Africa (ref 8), 23% had recorded VL in the range 51-999 copies/ml, but in most cases these were single measurement LLVs. The current study uses a stricter definition of LLV. Data on prevalence of repeat LLV in the study population could also affect the public health impact of regimen switch in LLV (as brought up in the discussion). Perhaps the first step is paying attention to VL in the LLV range, and not considering these as suppressed patients (as is currently the case) - and the proportion who have repeat LLV and need regimen switch is likely to be much lower.

***Response****: Thank you for this valid consideration. According to local and WHO guidelines, patients with LLV in Lesotho are considered as “suppressed” and thus they are not followed up with intensified adherence counseling and a follow-up VL after 2-3 months. Therefore, it is difficult to estimate the incidence/prevalence of persistent/sustained LLV used in our definition. However, Hermans et al. (ref 18; Lancet Infect Dis 2017) reported that among the 23% (N=16013) patients with LLV (51-999 copies/mL), 10% (n=1605) showed LLV at two consecutive measurements. The analysis was based on their cohort data over a period of 10 years. We adapted the “Discussion” section as follows:*

It is difficult to number precisely the amount of additional workload caused by lower VL thresholds. A recent cohort study from South Africa observed that over a period of 10 years among 69454 patients on first-line ART, 16013 (23%) had a single instance of low-level viremia (51-999 copies/mL) and among these, 10% (1605) showed LLV at two consecutive measurements [18]. These data suggest that particularly following up all first-time LLV would require a substantial amount of resources.
2.	For the analysis, virological failure was defined as VL>50 copies/ml at 24 and 36 weeks. I recommend including a reference for this definition and/or a motivation of why this definition of virological failure was chosen.

***Response****: As our trial compares the definition of virological failure in guidelines from Europe/US to the one used in WHO guidelines, we opted for 50 copies/mL in accordance with EACS guidelines.*

3.	Methods, page 9, row 205: " … narrower pre-defined primary endpoint visit windows …" - I would appreciate an explanation of how this was done.

***Response****: This sentence refers to supplement table 5, the pre-defined sensitivity analyses. Two sensitivity analyses were performed using narrower primary endpoint windows than what was used for the primary analysis. In order to clarify, we added reference to supplement 5 in the methods, and we adapted footnote 3 of supplement table 5.*

4.	 Participants in the study were on long-term ART (median 5.9 years). A comment could be added in the discussion that the implications of the study may not applicable during the first 12 months after ART initiation. 
***Response****: We added the following under limitations:*

Finally, it is noteworthy that the trial population has been taking first-line ART for a median duration of 5.9 years. Our findings may thus not be applicable to persons with LLV less than one year after ART initiation.

5.	The levels of adherence are overall surprisingly low. This could reflect a more accurate recording of true adherence (often overrated), but may be commented in the discussion. 
***Response****: We added the following comment in the “Discussion” section:*

Adherence assessments are difficult to compare across different trials, but the overall self-reported adherence in our trial appears to be lower than in the lopinavir/ritonavir treatment arms in two large second-line trials in Africa (Aboud et al. DAWNING trial, The Lancet Infectious Diseases, 2019 & Paton et al. EARNEST trial, NEJM, 2014). One reason may be that LLV was less perceived as an important problem among providers as well as patients and thus efforts to improve adherence were less intensive.

6.	Discussion, page 16-17: With regard to studies on regimen switch in patients with LLV, the authors could also mention a study by Boillat-Blanco et al (PMID: 24964403), with findings that support those of the current study. 
***Response****: We added the suggested reference.*

7.	In the discussion, it would be interesting to mention different mechanisms of LLV, and how this could impact the effect of ART modification. For example, the risk of selection of drug resistance (and subsequent virological failure) is probably higher in persons with LLV due to ongoing viral replication during ART, and such individuals are likely to benefit from regimen switch. On the other hand, this may not be effective in cases where LLV is due to release of virions from latently infected cells (monoclonal LLVs). This is discussed in reference 23, showing that ART modification in LLV was effective in cases with demonstrable drug resistance and/or poor adherence, but not in cases in which these factors were not observed.

***Response****: We thank the reviewer for this very valid point and agree that this is a very interesting question. We, however, feel that it would go beyond the scope of this manuscript that reports on the primary and secondary endpoints of the SESOTHO trial as per protocol.*
